# Transomental Hernia: Rare Presentation in a Virgin Abdomen

**DOI:** 10.7759/cureus.76126

**Published:** 2024-12-21

**Authors:** Asiyah Shafi, Fatema Karmustaji, Muhammad Umar Younis, Monis Ahmed, Roger Gergy

**Affiliations:** 1 General Surgery, Mohammed Bin Rashid University of Medicine and Health Sciences, Dubai, ARE; 2 General Surgery, Mediclinic City Hospital, Dubai, ARE

**Keywords:** internal hernia, intestinal obstruction, small bowel obstruction, surgical acute abdomen, transomental band, transomental hernia, virgin abdomen

## Abstract

Internal hernias are characterized by the protrusion of abdominal viscera through congenital or acquired apertures within the abdominal cavity and are a recognized etiology of intestinal obstruction. Internal hernias can cause symptoms ranging from mild abdominal discomfort to complete intestinal obstruction. Transomental hernias are often associated with postoperative anatomical changes and are rare in patients without prior abdominal surgeries. This report details one case of a 31-year-old otherwise healthy female with no history of prior surgery, highlighting the clinical variability associated with internal hernias.

The patient presented with severe epigastric pain and nausea, with initial ultrasound findings unremarkable. Hours later, she returned with left iliac fossa pain and vomiting. A CT scan of the abdomen with contrast showed free fluid and dilated bowel loops, suggestive of small bowel obstruction. Emergency laparoscopy revealed small bowel herniation through a congenital omental band and ischemia, which resolved after the band was released. The patient recovered uneventfully and was discharged on day three.

Transomental herniation exhibits nonspecific clinical symptoms of bowel obstruction. Radiographic presentations are generally nonspecific; however, a conclusive diagnosis is typically reached during surgery, where the detection of gangrenous bowel tissue is common, especially during exploratory laparoscopy. The postoperative mortality is largely attributed to bowel necrosis and delays in initiating treatment. This emphasizes the significant risk posed by undiagnosed cases and the critical importance of timely and effective intervention.

## Introduction

An internal hernia is defined as a protrusion of viscus through a mesenteric, omental, or peritoneal aperture within the peritoneal cavity, which can be congenital or acquired [1,2]. It is a rare situation that accounts for approximately 5% of all intestinal obstruction cases [3,4]. An internal hernia may manifest as one of various subtypes, with paraduodenal (53%) and pericecal (13%) hernias being the predominant types, followed by hernias through the foramen of Winslow (8%), transmesenteric (2%), and then transomental hernias (1%) [5]. A transomental hernia is the rarest subtype of internal hernia, and while it may occasionally be caused by a congenital abnormality, the majority of transomental hernias are attributed to iatrogenic or post-traumatic causes, including other causes related to peritoneal inflammation [3,4,6]. These usually present as an acute strangulation of the small bowel [7]. Spontaneous internal hernias in a patient without a background surgical history may present in the case of senile atrophy of the omentum. In other situations, however, discovering a transomental hernia in a patient with a virgin abdomen is exceedingly rare [4]. This is a rare yet critical case, as internal hernias are linked with high morbidity and mortality rates, thereby necessitating urgent diagnosis and surgical intervention. In this report, we have presented a rare case of spontaneous transomental hernia that presented as a small bowel obstruction in a patient with a virgin abdomen and that was subsequently discovered and treated successfully in our hospital.

## Case presentation

A 31-year-old previously healthy female presented to the emergency department (ED) with a chief complaint of sudden onset of abdominal pain and associated nausea. The patient denied any recent illness or preceding trauma. She had no significant medical, surgical, or social history.

The abdominal pain was described as sharp and constant, localized primarily to the epigastric region, with radiation to the left lower quadrant. No relieving or exacerbating factors were reported. Additionally, the patient complained of associated nausea but denied any vomiting, changes in bowel habits, or urinary symptoms. Upon initial evaluation, the patient appeared uncomfortable but was alert, oriented and hemodynamically stable. Abdominal examination revealed tenderness localized to the epigastric region, without distention, guarding or rebound tenderness. Bowel sounds were present in all quadrants, and there were no palpable masses or organomegaly noted. An abdominal and pelvic ultrasound was done without significant findings. The patient responded to symptomatic treatment and was discharged from the ED. 

**Figure 1:**
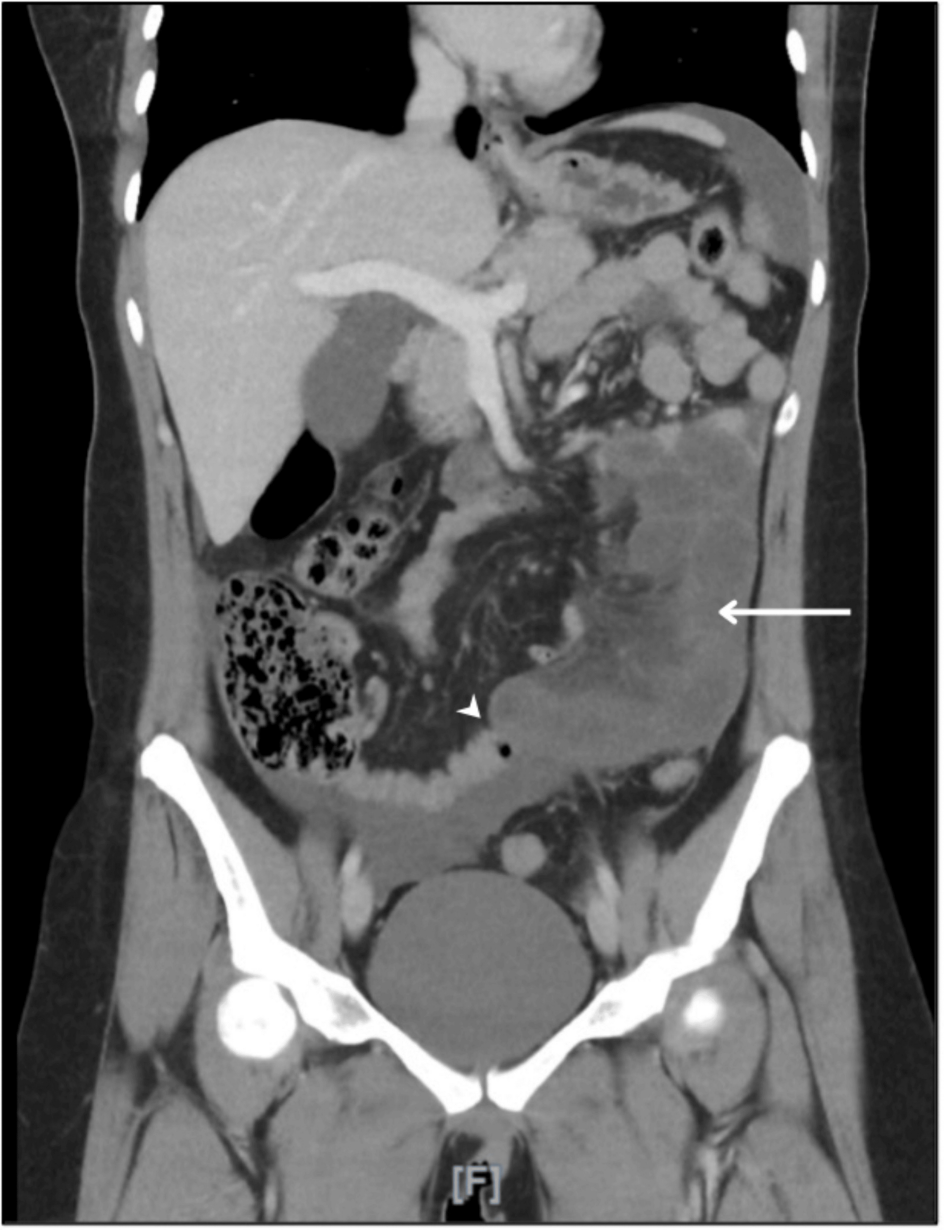
A contrast-enhanced CT scan of the abdomen reveals dilated intestinal loops on the left side (indicated by the arrow), with decreased contrast enhancement. The arrowhead points to the cut-off point of the congested bowel loops.

However, she returned a few hours later with multiple episodes of vomiting and remarkable tenderness in the lower left iliac fossa. A CT scan with contrast of the abdomen and pelvis was performed, revealing highly attenuated free fluid in the abdomen suggestive of hematoperitoneum. Dilated small intestinal loops were observed distally on the left side with reduced contrast enhancement compared to the proximal loops, as shown in Figure 1, suggesting possible bowel ischemia or obstruction. The images were reviewed by radiologists and the surgical team, revealing evidence suggestive of bowel obstruction secondary to an internal hernia. The patient was immediately admitted for an emergent exploratory laparoscopy.

**Figure 2:**
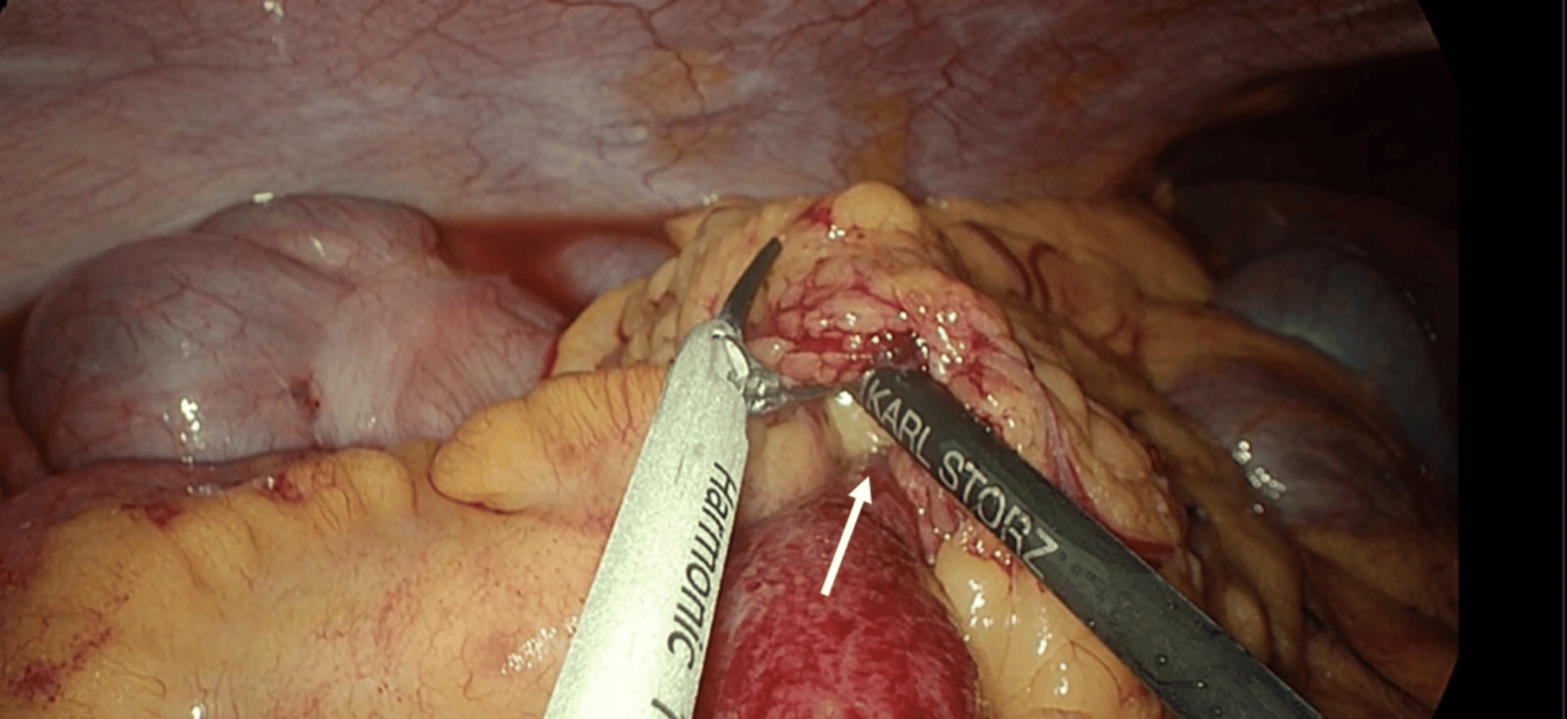
Herniating bowel through the omental band released from the posterior abdominal wall.

**Figure 3:**
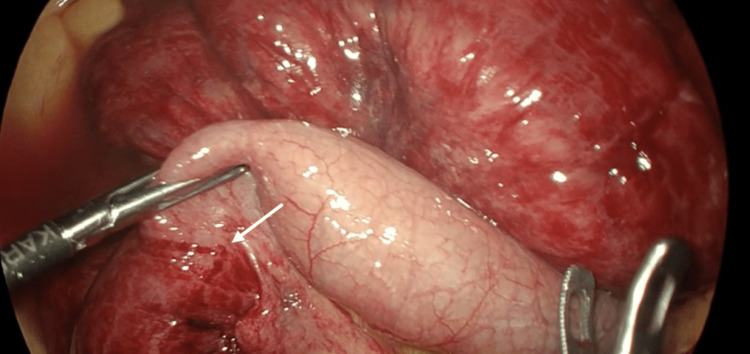
Congested bowel loops after being released from hernial sac and proximally the unaffected bowel. Line of demarcation showing the point of obstruction.

Intra-operatively, the cause of the bowel obstruction secondary to internal hernia was discovered. A transomental band was found fixed to the posterior abdominal wall, causing herniation of the small bowel through a pocket, as shown in Figure 2. The omentum was divided, and the affected bowel was released. Approximately 60 cm of mid-ileal bowel was affected. Figure 3 shows both the affected and unaffected small bowel and illustrates a clear line of demarcation at the point of obstruction. A peritoneal lavage was performed, and a 20-minute break was taken to allow the affected bowel to recover. With reassuring signs of improved bowel perfusion, the surgery was concluded. The patient recovered well postoperatively and was discharged after three days. She was followed up as an out-patient and remains well to date without any complications. 

## Discussion

An internal hernia is a protrusion of viscus through a congenital or acquired mesenteric, omental, or peritoneal aperture within the peritoneal cavity. An internal hernia is a rare situation that may manifest as one of several subtypes, together representing approximately 5% of all cases of intestinal obstruction [3,4]. A transomental hernia is considered the rarest subtype, accounting for about 1% of internal hernia cases [5]. 

The majority of transomental hernias in adult patients emerge after the age of 50 and are often attributed to iatrogenic or post-traumatic causes, including other causes related to peritoneal inflammation [3,4,8,9]. Procedures such as Roux-en-Y gastric bypass, choledocojejunostomy, and gastrectomy are linked with increased susceptibility to developing a transomental hernia [10]. Occasionally, a spontaneous transomental hernia may develop in a patient without a background surgical history, usually associated with senile atrophy of the omentum. Discovering a transomental hernia in other cases of a virgin abdomen is a rarer occurrence [3,4,8,9]. 

The absence of a hernia sac and a small orifice size are features that distinguish a transomental hernia from other subtypes of internal hernia. These features also increase the tendency for strangulation of a transomental hernia compared to other subtypes [9]. Postoperative mortality rates can be as high as 30%, making heightened clinical suspicion crucial for early diagnosis and consequently reducing morbidity and mortality rates [3,4,8-10].

Preoperative diagnosis of a transomental hernia is often challenging because clinical symptoms are non-specific and similar to those of other causes of acute bowel obstruction, including abdominal pain, nausea, vomiting, abdominal distention, and constipation [3,4,8,9]. Abdominal CT imaging may play a key role in diagnosing transomental hernia in the presence of certain characteristic signs. One such sign is the “beak sign,” representing a triangle-shaped transition zone between a dilated proximal loop and a herniated bowel segment [3,4,8,9]. The “beak sign” may also alternatively be seen between the herniated dilated loop and collapsed distal loop. Another characteristic sign visualized on CT imaging is the “whirl sign,” which describes a swirling pattern of engorged mesenteric vessels accompanied by bowel wall thickening. Additionally, a transomental hernia may be suspected when dilated loops of bowel are located within the lesser sac [3,4,8,9]. Nonetheless, radiographic presentations are generally considered non-specific as well. In a series of 49 surgically diagnosed cases of internal hernias, only 16% of preoperative CT scans were considered suspicious [9]. In most cases of transomental hernia, a definitive diagnosis is made intraoperatively [3,4,8,9]. 

Surgical treatment of a transomental hernia involves reduction of the herniated bowel loops. If irreversible ischemia, necrosis, or perforation of the herniated bowel segment is present, resection of bowel becomes necessary. The omental defect is thereafter repaired to prevent recurrence of herniation [3,4,8,9]. Repair is performed through closure by suture, division, or in some instances, partial omentectomy. Surgical treatment may be performed through laparotomy or laparoscopy in select patients. Recent improvements in surgical techniques have allowed laparoscopic treatment of transomental hernia to be carried out safely, achieving minimal invasiveness without evidence of necrosis or perforation [3,4,8,9]. Most reported cases treated laparoscopically involve the release of incarcerated bowel, while cases involving difficult-to-assess bowel viability are treated through laparotomy [11]. No gold standard guideline is currently established for evaluating bowel viability, particularly in laparoscopic surgery [11]. One case report suggests that laparoscopic surgery can be performed safely in cases of internal hernia, including those with borderline or severe bowel viability, by adding a small laparotomy to confirm bowel viability [11].

Compared to other types of internal hernias, patients with transomental hernias are more likely to present with small bowel strangulation [3,4,8,9]. In many cases, exploratory laparotomy reveals gangrenous bowel. Consequently, transomental hernias have a high postoperative mortality rate of 30%, making prompt diagnosis and emergency treatment essential [3,4,8-10].

## Conclusions

Bowel obstruction can present with varied etiologies and may require a systematic approach to evaluation and management. This case highlights the importance of maintaining a high index of suspicion of rarer etiologies of internal hernias, such as congenital bands, in patients presenting as a case of bowel obstruction, particularly those who have a virgin abdomen. Timely diagnosis and intervention are crucial for optimizing patient outcomes and preventing morbidity and mortality. Close collaboration between emergency physicians, radiologists, and surgeons is paramount for prompt diagnosis and appropriate intervention in cases of bowel obstruction secondary to internal hernia in patients who have never undergone surgery.
